# High Power UV-Light Irradiation as a New Method for Defect Passivation in Degraded Perovskite Solar Cells to Recover and Enhance the Performance

**DOI:** 10.1038/s41598-019-45756-1

**Published:** 2019-07-01

**Authors:** Farzaneh Arabpour Roghabadi, Nasibeh Mansour Rezaei Fumani, Maryam Alidaei, Vahid Ahmadi, Seyed Mojtaba Sadrameli

**Affiliations:** 1 Optoelectronic and Nanophotonic Research Group, Faculty of Electrical and Computer Engineering, Tarbiat Modares University, Tehran, Iran; 2 Faculty of Chemical Engineering, Tarbiat Modares University, Tehran, Iran

**Keywords:** Devices for energy harvesting, Solar cells, Solar energy and photovoltaic technology

## Abstract

Although the power conversion efficiency (PCE) of perovskite solar cells (PSCs) reached up to 23%, their short lifetime and fast degradation still remain as the main challenges. In this work, a new facile optical method based on the high power UV-irradiation is presented for the recovery of the degraded PSCs. Addition to the full recovery of the performance, about 20% PCE enhancement and hystersis reduction are also achieved by UV-irradiation. UV-treatment causes modifications in both the bulk properties of the perovskite layer and the energy equilibrium at the interfaces. It is shown that UV-treatment effectively passivates the surface and grain boundaries defects in different types of the devices comprising normal and inverted configurations that is confirmed by the reduction of the density of defect states (DOS). It is proposed that UV-light passivates the shallow and deep defects by dissociation of adsorbed hydroxyl groups and water molecules during the device storage.

## Introduction

Organic-inorganic perovskite materials encompass a unique set of exciting optical and electrical attributes including high absorption coefficient^1^, tunable bandgap^2^, long intrinsic carrier lifetime^3^, high and balanced charge carrier mobility^4^, long charge carrier diffusion length^5^, and achieving impressive PCEs up to 23% with a relatively facile and low-cost fabrication methods. Although the PCE of perovskite solar cells has rapidly risen up to 23%, the significant challenge still remains regarding the device stability and lifetime^6^. In the meantime, the stability of the perovskite devices is a vital factor that defines their feasibility of commercial production. It is known that the degradation and lifetime of a device are strongly dependent on all its components, fabrication process, and storing conditions^7,8^. In the case of the complete device degradation, several reasons are expressed for perovskite solar cell degradation such as the degradation of each device component^9,10^, the UV irradiation, chemical reaction between the perovskite layer and metal contacts, water molecules diffusion^11–14^, and the surface and grain boundaries defects^15^. These defects originate from the low thermal stability, or low formation energy of perovskite materials that cause instability issues, comprising ion migration and associated current hysteresis that lead to the degradation of the device^15^. It was reported that the degradation of the perovskite grains was started from the defective surfaces because of the small migration activation energy of ionic defects such as iodine and methylammonium vacancies. Defects could also accelerate the degradation of the perovskite devices by initializing the diffusion of oxygen and water molecules into the perovskite materials^15,16^. Several strategies have employed to prevent or delay the degradation of perovskite solar cells such as encapsulation, doping^17^, defect passivation^15^, and inserting appropriate electron or hole transporting layers^18,19^. In spite of numerous efforts taken to prevent the device degradation, performance loss is observed in all devices. Further, in almost all studies, UV light was introduced just as a degradation reason for solar cells^12,20^. Nie *et al*. suggested that the photocurrent degradation in solar cell is induced by light soaking^21^. Leijtens *et al*. reported that under UV light irradiation, both encapsulated and unencapsulated perovskite devices showed the performance reduction however the encapsulated device degradation is more significant. It was attributed to the trapped photoelectrons caused by the formation of oxygen desorption surface states in the TiO_2_ surface which were passivated in the presence of oxygen. Therefore, because of the decreasing of O_2_ molecules diffusion into the encapsulated device, the passivation possibility reduced, led to the fast performance reduction^12^.

In this work, in spite of other works that reported the degradation effects of UV on the device performance and its component, the performance enhancement and full recovery of the degraded perovskite devices are achieved by a high power UV-irradiation at a short time. The performance of different types of devices including normal configuration (FTO/bl-TiO_2_/mp-TiO_2_/CH_3_NH_3_PbI_3_/P3HT/Au), inverted configuration (ITO/PEDOT:PSS/CH_3_NH_3_PbI_3_/PCBM/Ag), and hole transporting material-free (HTM-free) device (FTO/bl-TiO_2_/mp-TiO_2_/CH_3_NH_3_PbI_3_/Au) are recovered. To find the exact mechanism of the recovery, the normal device is completely analysed.

## Results and Discussion

**Figure 1:**
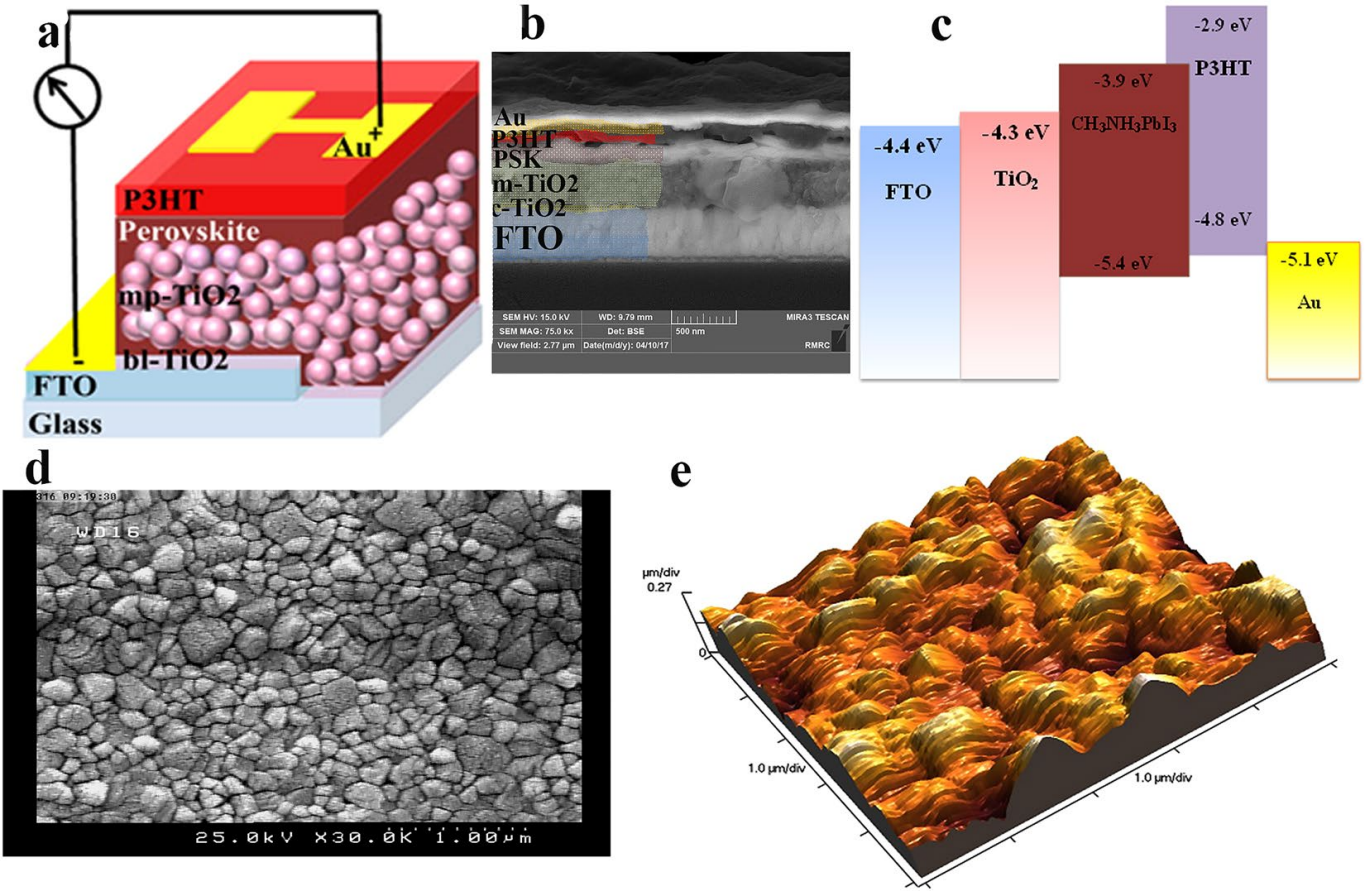
(**a**) Schematic structure of the normal perovskite solar cells. (**b**) SEM image of the cross section of the normal perovskite devices. (**c**) Schematics of the energy diagram of the normal device components. (**d**) SEM image of the perovskite layer. (**e**) Topography image of the perovskite layer.

**Figure 2:**
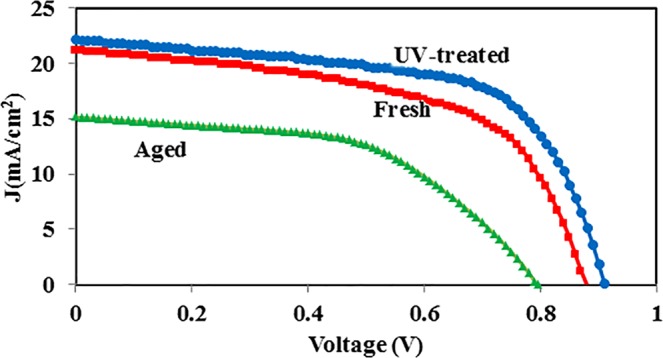
J-V characteristics of the fresh normal device and the aged normal device before and after UV-treatment.

**Figure 3:**
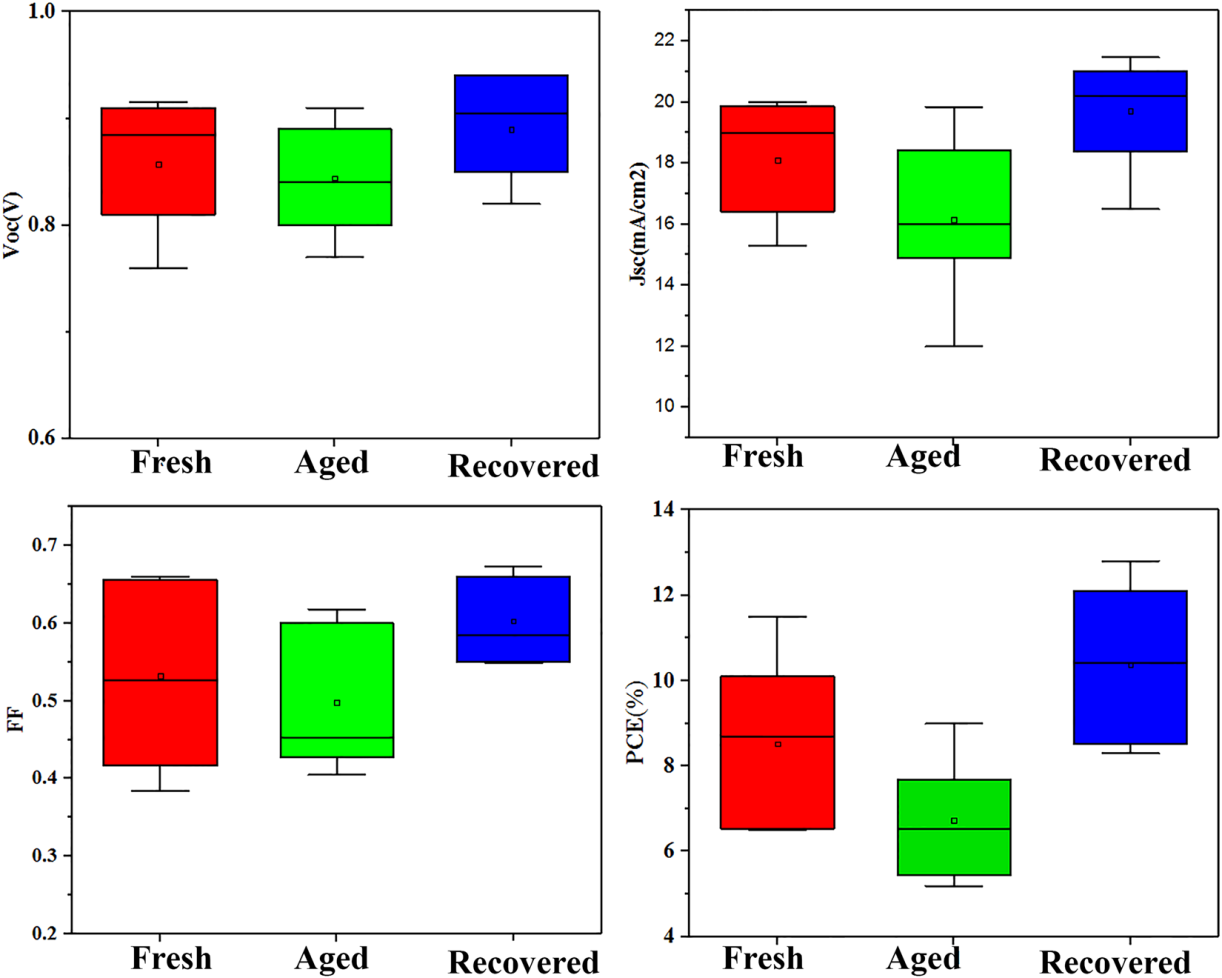
Statistical analysis of the photovoltaic performance of the fresh, aged and recovered devices by 4 min UV-irradiation.

Figure 1 shows the structure, SEM cross section image and energy diagram of the normal perovskite device (FTO/bl-TiO_2_/mp-TiO_2_/CH_3_NH_3_PbI_3_/P3HT/Au). The morphology and topography images of the perovskite layer are exhibited in Fig. 1d,e. The root mean square (RMS) of the perovskite layer is estimated to be around 70 nm. The J-V characteristic of one of the aged devices before and after UV-treatment (more details in Supplementary Information) for 4 min under 1-sun illumination is shown in Fig. 2. As summarized in Table 1, the *PCE* of the normal device decreases from 10.5% to 6.3% during the storage in the ambient condition for 35 days. By employing UV- irradiation, the PCE of the aged device increases from 6.3% to 12.6%. For better presenting of the recovery of the devices, the statistical analysis of the performance parameters is exhibited in (Fig. 3). It reveals that the performance of the degraded devices not only is fully recovered, but also the recovered PCEs are higher than the initial one. To select the optimum time duration for treating, normal devices are treated with different exposure time between 30 s to 10 min under UV-light with a power of 400 and 1000 W. It is resulted that a 1000W-UV light provides better recovery (Table S1) and 4 min is the optimum time that leads the best performance. Interestingly, the hysteresis of the normal device reduces after the UV-treatment (Fig. S1). To define the dependency of the UV-treatment recovery on the device structure, inverted devices with ITO/PEDOT:PSS/CH_3_NH_3_PbI_3_/PCBM/Ag structure (Fig. S2) and HTM free devices are also examined. As summarized in Tables S2 and S3, HTM-free device and inverted device are also recovered after UV-treatment. It should be noted that, the UV-treatment of the fresh devices also shows performance improvement but not as much as the improvement observed for the stored devices (Table S4).

**Table 1:** Photovoltaic performances of fresh, aged, and recovered normal devices.

Device	V_OC_ (V)	J_SC_ (mA/cm^2^)	FF (%)	PCE (%)
Fresh device	0.88	21.19	56.2	10.5
Aged device	0.79	15.14	52.9	6.33
Treated device	0.92	22.21	61.5	12.58

**Figure 4:**
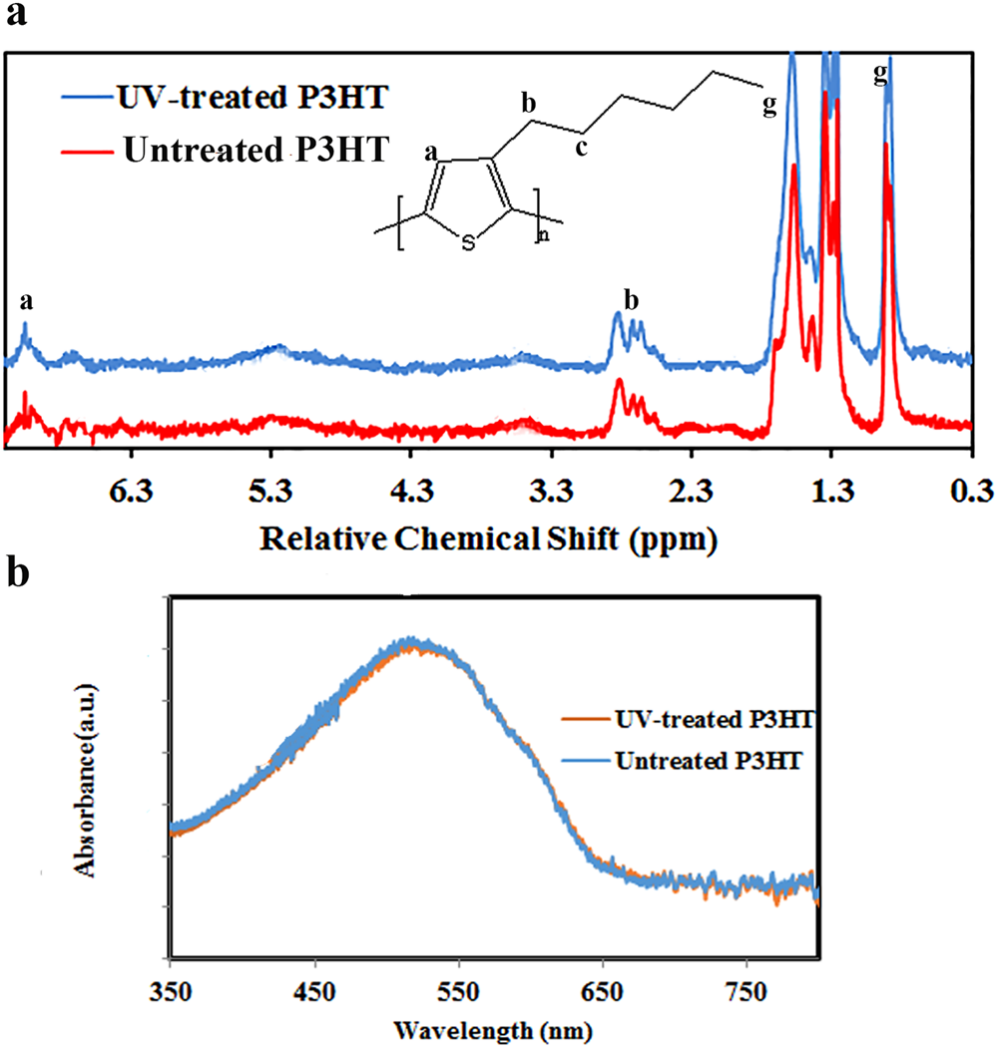
(**a**) NMR spectra of P3HT, and (**b**) absorption spectra of P3HT layer before and after UV-treatment for 4 min.

To understand the possible effects of UV-treatment, the response of the normal device components to the UV light is investigated. As mentioned, UV light was defined as the main function for device and its layers degradation. P3HT used as HTM in the device is one of the main candidates to be degraded during UV-treatment. According to H-NMR spectra of fresh P3HT and exposed P3HT into the UV light, during the UV irradiation for 4 min, no changes happen in the structure (Figs 4 and S3). While, some structural changes are observed in P3HT film treated under UV light for 10 min. It means that the P3HT degradation does not occur during the 4 min-treatment. Likewise, no difference is observed between the absorbance spectrum of P3HT before and after UV treatment (Fig. 4b).

The absorbance spectrum of bl-TiO_2_/m-TiO_2_ double layer utilized as ETL is exhibited in Fig. S4. The obtained optical band gap for both of the TiO_2_ layers (before and after treatment) is around 3.1 eV. As a result, the band gap value of TiO_2_ layer before and after UV-treatment does not change. But, the higher absorption tail is observed for the treated layer that indicates a higher density of defect states after UV-treatment^22^. The XRD diffraction peaks of CH_3_NH_3_PbI_3_ absorber layer deposited on FTO and FTO/TiO_2_ substrates before and after UV-treatment are exhibited in Fig. S5. CH_3_NH_3_PbI_3_ is deposited on the ETL layer to record the possible interactions in the interfaces. Simultaneous enhancement of the intensity of the peak located at 2θ = 12.7° signed as PbI_2_ plane and reduction of the intensity of the perovskite peak at 2θ = 14.15° reveals the decomposition of some of the perovskite crystals during UV exposure in the perovskite single layer deposited on FTO and FTO/TiO_2_ substrates (Fig. S5b). This decomposition is confirmed by the observed decrease in the UV-visible absorbance of the layer (Fig. S5b). Besides, the absorption edge of the perovskite films is not affected by UV-light that reveals no change for the band gap of perovskite layer. The photoluminescence spectra (PL) of the perovskite layer before and after treatment are presented in Fig. S5. It is expected that because of the decomposition of the crystals and enhancement of the amount of PbI_2_ during the treatment, PL intensity reduces. While, the intensity is not changed significantly and the FWHM increases slightly (Fig. S6). Figure S5e shows the FTIR spectra of CH_3_NH_3_I as the precursor and the perovskite films before and after UV-treatment. When the MAI molecule reacts with PbI_2_ and perovskite forms, almost all MAI peaks are shifted to other frequencies because of the change in the force constant of the bonds. The absorption peaks at 2961 and 1404 cm^−1^ are an asymmetric stretch and an asymmetric bend of CH_3_ of MAI^14^, respectively. Also, the broad peak at 3095 cm^−1^ is related to the symmetric and asymmetric stretching modes of NH_3_. In addition, the MAI peak at 990 cm^−1^ is indexed to C-N stretching mode^14^. Comparison of the FTIR peaks before and after treatment reveals that during the treatment, no extra peak is appeared in the perovskite film after UV-irradiation. It indicates that in the perovskite single layer, crystals are decomposed into PbI_2_ and CH_3_NH_3_I through the reaction 1 and also if the CH_3_NH_3_I molecules are dissociated, the residual chemical components have gas phase and leave the solid film. As known the perovskite crystals decompose through the proposed reversible reactions presented below^23^:

1 $${{\rm{CH}}}_{3}{{\rm{NH}}}_{{\rm{3}}}{{\rm{PbI}}}_{3}({\rm{s}})\leftrightarrow {{\rm{CH}}}_{3}{{\rm{NH}}}_{3}{\rm{I}}({\rm{aq}})+{{\rm{PbI}}}_{2}({\rm{s}})$$

2 $${{\rm{CH}}}_{3}{{\rm{NH}}}_{3}{\rm{I}}({\rm{aq}})\leftrightarrow {{\rm{CH}}}_{3}{{\rm{NH}}}_{2}({\rm{aq}})+{\rm{HI}}({\rm{aq}})$$

**Figure 5:**
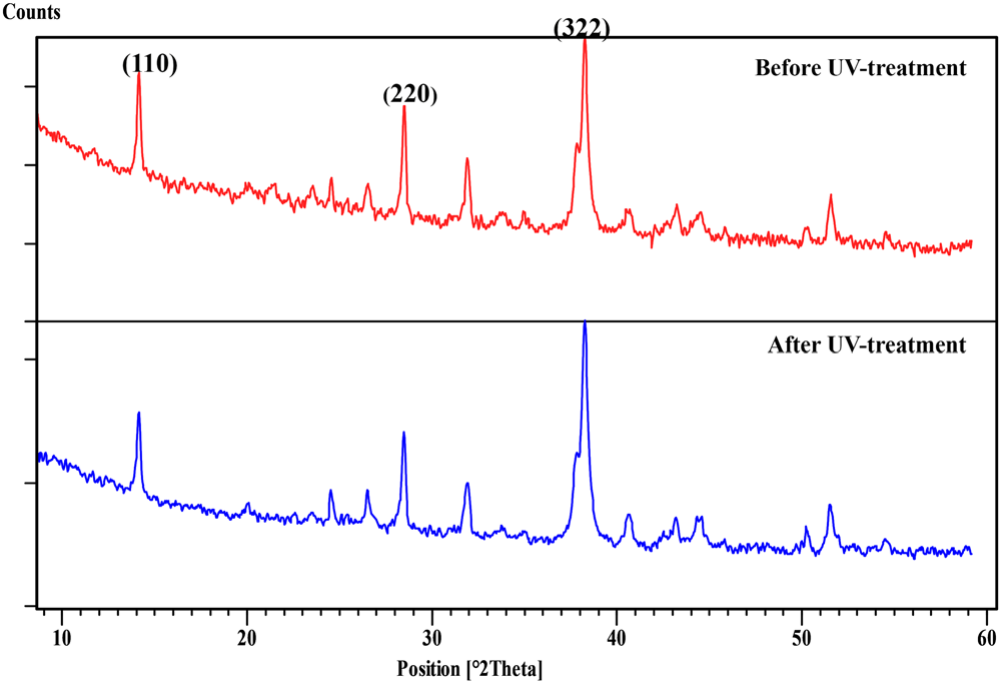
XRD spectra of complete device with FTO/c-TiO_2_/m-TiO_2_/CH_3_NH_3_PbI_3_/P3HT/Au structure before and after UV-treatment.

In contrast to the FTO/CH_3_NH_3_PbI_3_ and FTO/bl-TiO_2_/m-TiO_2_/CH_3_NH_3_PbI_3_ multilayers, perovskite crystals in the FTO/bl-TiO_2_/m-TiO_2_/CH_3_NH_3_PbI_3_/P3HT multilayer and complete device (FTO/bl-TiO_2_/m-TiO_2_/CH_3_NH_3_PbI_3_/P3HT/Au) are stable during the UV treatment (Figs 5, S7). It seems that, when P3HT hole transporting layer is present upon the perovskite layer, decomposition of the perovskite layer does not occur. Methylamine and hydroiodic acid are the products of perovskite material decomposition that both exist in the gas phase at room temperature because of their low boiling points (−6 °C and −34 °C for HI and methylamine, respectively)^14^. As a result, in the open system, they continuously release and drive the decomposition reaction forward (Reactions 1 and 2). It can say that the decomposition of the perovskite layer in FTO/CH_3_NH_3_PbI_3_ and FTO/bl-TiO_2_/m-TiO_2_/CH_3_NH_3_PbI_3_ is according to these reactions. But, in the completed device and FTO/bl-TiO_2_/m-TiO_2_/CH_3_NH_3_PbI_3_/P3HT multilayer the perovskite layers are covered by P3HT/Au and P3HT that can prevent the releasing of methylamine and HI as gas molecules and also CH_3_NH_3_PbI_3_ molecules trapped in the grains during crystal formation.

**Figure 6:**
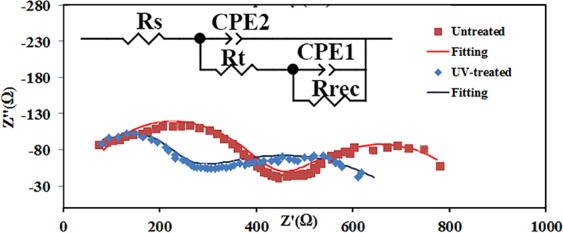
Impedance spectra of the device before and after UV treatment under open circuit condition.

To understand the electrical changes happen in the device after the UV treatment, the impedance spectra of the device before and after the treatment are recorded under light at open circuit condition. To extract the device parameters, the spectra are fitted by the equivalent circuit presented in Fig. 6. In this model, the high frequency arc is fitted by resistance R_t_ coupled to capacitance C_2_. The low frequency arc is fitted by *C*_1_ and *R*_*rec*_ that represent the parameters associated with the contact to the perovskite. Capacitances and resistances extracted by fitting the equivalent circuit model are summarized in Table 2. The main change after UV-treatment is the significant reduction of R_3_. Guerrero *et al*. reported that R_3_ may in the standard picture correspond to the conductivity of the bulk perovskite, but it is heavily influenced by the contact transport resistance^24^.

**Table 2:** Extracted resistance and capacitance from the impedance spectra.

Device	R_t_ (Ωcm^2^)	R_rec_ (Ωcm^2^)	C_2_ (Fcm^−2^)	C_1_ (Fcm^−2^)
Untreated device	48.631	48.686	2.5E-07	2.4E-04
Treated device	17.523	60.5	1.2E-07	6.71e-5

C_2_ which is due to the diverse dipolar mechanisms such as CH_3_NH_3_^+^ or PbI_6_ octahedra reorientation^24^, and cooperative ionic off-centering, decreases after UV treatment. It means that the charges are transported more efficient after UV-treatment that causes the reduction of the charge accumulation. The low frequency capacitance (C_1_) is associated to the electronic and ionic accumulation at the electrode interfaces^24^. Further, the recombination resistance (R_rec_) of the treated device is higher than the untreated one. But, it can be concluded that the modification occurs in the device during the treatment reduces the recombination rate which is consistent with the obtained lower C_1_.

As previously mentioned, Lee *et al*. reported the UV-irradiation as the main factor for degradation of perovskite device and light soaking as the recovery function of the performance^20^. In their investigation, decomposition of perovskite layer and formation of PbI_2_ was determined as the reason for the recovery of the device after light soaking. It was argued that traps are formed during UV-irradiation and simultaneously passivated and neutralized by PbI_2_ ^20^. The recovery achieved in our work can’t be explained according to this proposed mechanism because XRD analysis (Fig. 5) reveals that in the complete device, decomposition of perovskite layer and formation of more PbI_2_ after UV-treatment do not occur.

In some reports, the degradation of the device under UV light was mainly attributed to the interaction of TiO_2_ layer into the UV irradiation. As reported, upon UV exposure, the photogenerated holes reacts with the oxygen radicals adsorbed at surface oxygen vacancies, leads to formation of deep surface trap sites that can trap electrons and enhance the recombination^12^. In our work, the device is illuminated from the Au contact side and around 15% of the incident UV light will insert into the device (Figs S8 and S9). Because of the high energy of the photons, it can be assumed that a high amount of the incident UV light is absorbed by P3HT and perovskite layer before it enters into the TiO_2_ layer. Therefore, observing no destructive effect of UV on TiO_2_ layer can be acceptable.

**Figure 7:**
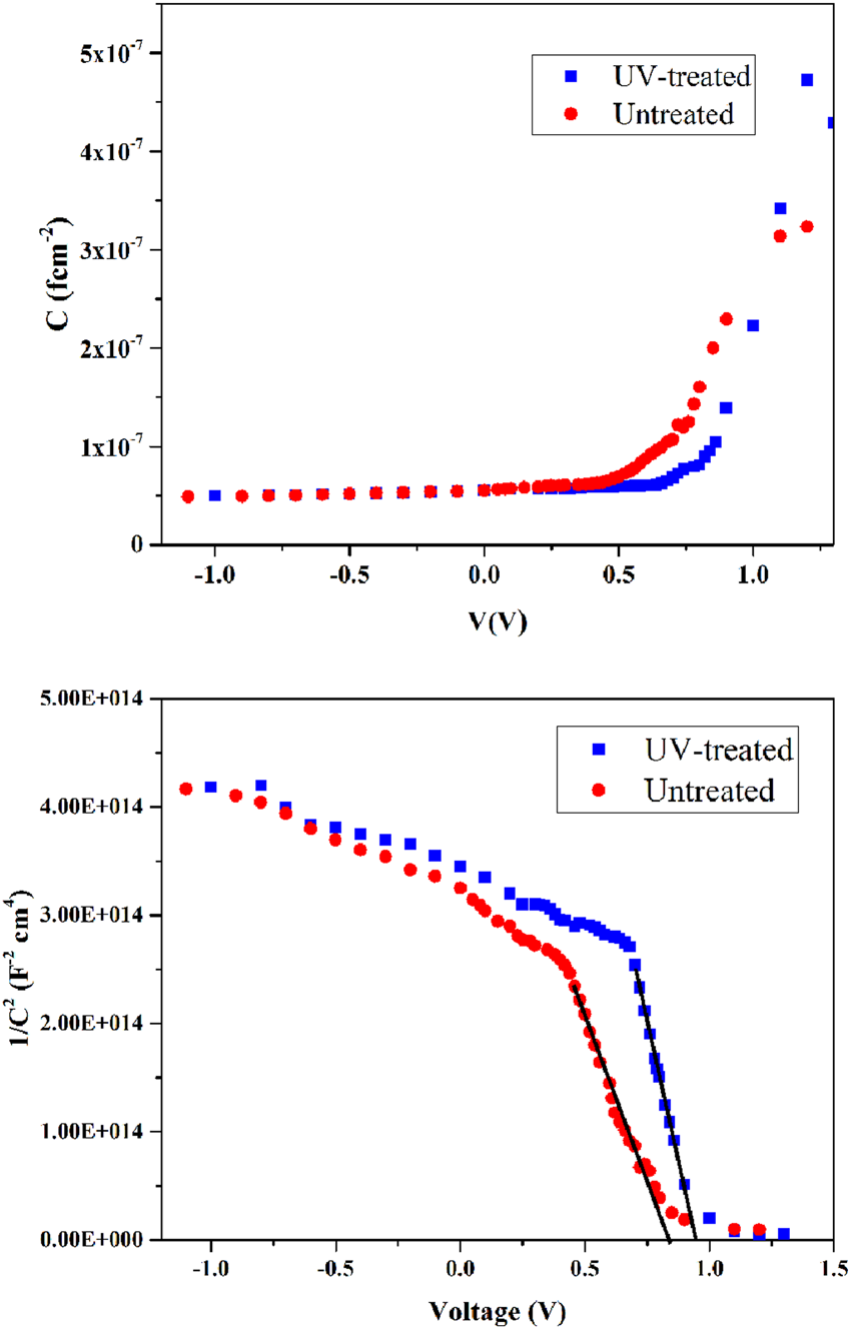
Capacitance−voltage measurements of perovskite device before and after UV-treatment carried out at 10 kHz. (**b**) Mott–Schottky characteristics *C*^−2^*(V)* of the device before and after UV-treatment.

The *C(ƒ)* curves of the device before and after UV-treatment are presented in Fig. S10 that show the ascending trend of the capacitance toward the low frequencies due to the contribution of the defect states in the gap. Defect states inside the bandgap contribute to the capacitance of the junctions depending on their energy and space position^25^. C-V measurment can be used to distinguish effects occur at the bulk of perovskite and those occur at the intefraces. Because, the doping densities and flat-band potential (V_fb_) depend on bulk properties of the layer and energy equilibration at the contacts, respectively^26^. The C-V characteristics of the device before and after UV-treatment are presented in Fig. 7. The intermediate capacitance observed in Fig. 7a defins the width of the deplation zone, corresponding to a Schottky barrier at the cathode. Full deplation conditions occur at reverse bias that correspounds to the geometrical capacitance (C_g_ = ***εε***_0_/***d***, where ***ε*** is the relative dielectric constant of the perovskite material, ***ε***_0_ is the permittivity of the vacuum, and *d* is the thickness of the active layer) determined by dielectric constact of the active layer. The relative dielectric constant measured from the C-V plot of the device is 19. The C^−2^(V) curves of the device before and after UV-treatment are presented in Fig. 7b. The measurement is done at the frequency which coincide with the central capacitance plateau observed at zero voltage linked to the dielectric response of the absorber^27^. The absorber defect density (N) and the flat-band potential (V_fb_) can be derived using Mott-Schottky (MS) relation^27^ (Eq. 1):

3 $$\frac{1}{{{\boldsymbol{C}}}^{2}}=\frac{2({{\boldsymbol{V}}}_{{\boldsymbol{fb}}}-{\boldsymbol{V}})}{q{\boldsymbol{\varepsilon }}{{\boldsymbol{\varepsilon }}}_{0}{\boldsymbol{N}}}$$

where *q* is the element charge. The slop of the straight line is related to the measurable density of electrical defects. The obtained defect densities for device before and after treatment are 1.3 × 10^16^ and 7.35 × 10^15^ cm^−3^, respectively, that shows the reduction of defect density after the UV-treatment. It suggests that the bulk properties of perovskite active layer are modified. Further, MS plot deviates from the linear dependence as bias approaches the flat-band potential in the forward direction (Fig. 7b). It exhibits an increase in *V*_*fb*_ of the device after the UV-treatment from 0.85 V to 0.94 V, indicating a modification in the energetic of all the elements involved at the cathode interface. It means that an accumulation of minority carriers reduces and causes a decrease of the quasi-Fermi level and an additional charge in the surface state. The reduction of these additional charges at the surface state leads to a displacement of the apparent flat-band potential^28^.

**Figure 8:**
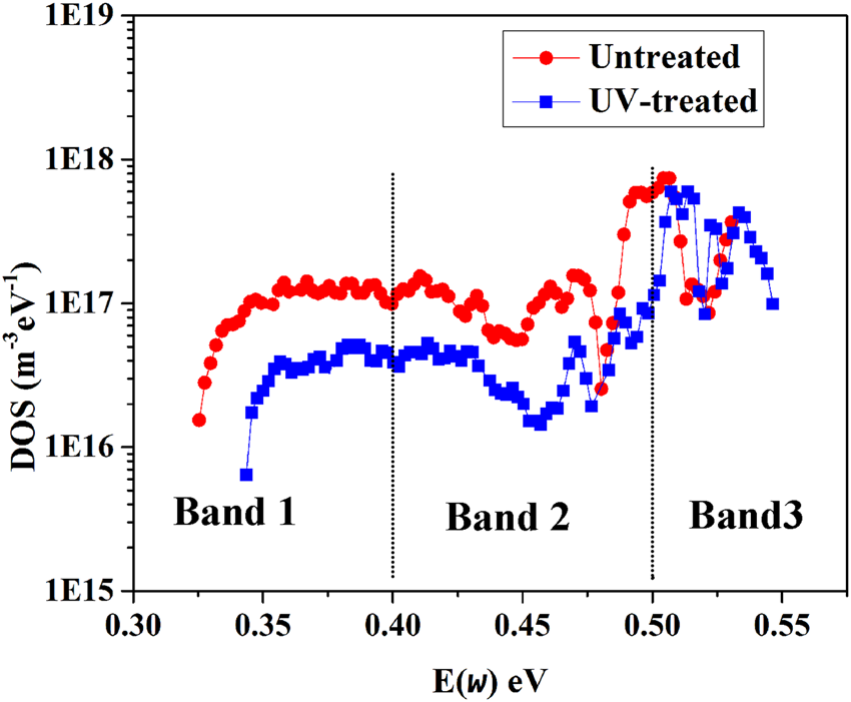
Trap density of stats (DOS) of perovskite device before and after UV-treatment.

As known, at low angular frequency of the perturbation, the capacitance reveals the energy density of defect states (DOS) in the depletion region^25^. The trap density of states (DOS) can be obtained from the angular frequency dependent capacitance employing the Equation 2 ^29^:

4 $${N}_{T}({E}_{\omega })=-\,\frac{{V}_{fb}}{qW}\frac{dC}{d\omega }\frac{\omega }{K{T}_{B}}$$

where *ω* is the angular frequency, T is the temperature, and K is the Boltzmann constant. Further, W is the depletion width that is extracted from Mott-Schottky analysis. An energetic demarcation (*Eω*) is defined using angular frequency (Eq. 3),

5 $${E}_{\omega }={K}_{B}Tln(\frac{{\omega }_{0}}{\omega })$$

where *ω*_0_ is the attempt-to escape frequency that was estimated to be ~2 × 10^11^ for dominant traps^30^. The defect stats (DOS) obtained from C-ƒ spectra of the device at room temperature are exhibited in Fig. 8. The DOS with an energy level reduces after the UV-treatment of the device. Trap bands with different trap energy depths can be defined in defect density profile. The DOS in Band 1 (<0.4 eV) is attributed to the shallow trap states that is smaller in the UV-treated device. The density of shallower trap states is attributed to traps at grain boundaries^15^ that are passivated by UV-irradiation. Further, the treated device shows a lower DOS in the deeper trap region (0.4–0.52 eV) that is related to the defects at the surface^15,29^. It indicates the effective passivation of shallow and deep charge traps in the perovskite device that reduces the carrier scattering and trapping in the defects. It should be noted that, the significant reduction of charge transport resistance observed in EIS analysis after the treatment can be attributed to this defect passivation during UV-treatment. As explained, charge transport resistance significantly reduces after the UV-treatment. Passivation of the defects that introduce deep levels in the band gap can be the reason for the reduction of R_tr_ because they are effective carrier traps and non-radiative recombination centres^31^. Surface defects would dramatically impact the *V*_OC_ of the devices because of their energy disorder and decreased carrier concentration by pulling down the quasi-Fermi level splitting^15^.

### The proposed recovery mechanism

**Figure 9:**
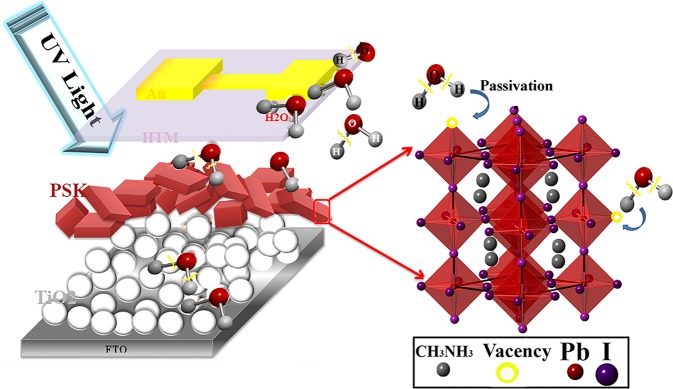
The proposed mechanism of the UV-assisted recovery and performance enhancement.

As stated, Mott-Schottky analysis and DOS of the device reveal that the observed remarkably recovery and performance enhancement after UV-treatment can be attributed to the both shallow and deep defects passivation under UV irradiation. The native point defects include vacancies, interstitials, and antisites that play an important role in carrier trapping and recombination^31^. An anionic Pb–I antisite defect on a PbI_2_-terminated surface and a cationic Pb cluster on a MAI-terminated surface were defined as two surface defect sites to generate deep charge traps^15^. Thus, the achieved improvement can be attributed to the passivation of these defects during the treatment. It was known that during the formation and storing of the perovskite layer, halides and cations are lost from the perovskite crystal resulting in under-coordinated Pb atoms both on the surface and grain boundaries of the film and vacancy sites are formed that act as electronic trap sites^32^ (Fig. 9).

In silicon solar cells, defect passivation of Si is mostly performed by the elimination of the Si dangling bonds by formation of Si-H, Si-N, or Si-O covalent bonds^33^. Vidhya *et al*., improved the silicon solar cell performance using atomic hydrogen and oxygen activated by the breakdown of water molecules by laser heating to passivate the dangling bonds in the crystal grains^34^.

**Figure 10:**
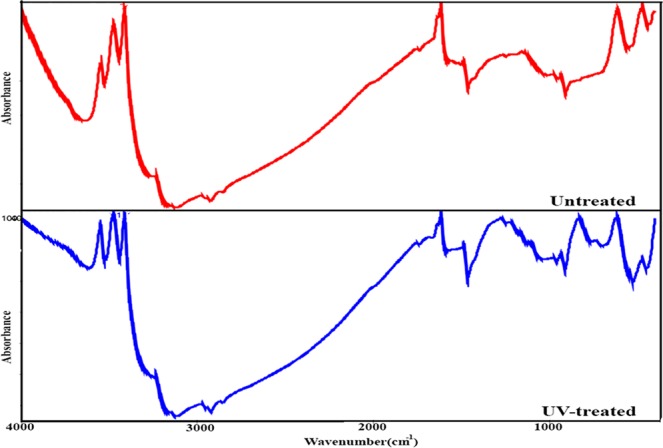
FTIR spectra of complete device before and after UV-treatment.

Our proposed mechanism for the shallow and deep defect passivation of UV-treatment is attributed to the events that happen in the device. Because the complete device is illuminated from the metal contact side. It should be noted that the illumination from FTO side also shows desired effect however is not as efficient as illumination from cathode side. It seems that the fraction of UV-light that passes from the Au contact and inserts into the perovskite layer, passivates the surface defects and grain boundary defects by dissociating adsorbed hydroxyl groups and water molecules in the device. Due to the fabrication and storing of the devices in the ambient condition, the presence of superficial hydroxyl groups cannot be neglected at the interfaces and the bulks that have strong effect on the device performance^35,36^. Further, it was confirmed that the surface of TiO_2_ can react immediately with water molecules in either aqueous solutions or humid air, leads to formation of hydroxyl groups that are adsorbed physically or bound chemically^37^. In the FTIR spectra of the complete device (Fig. 10), the observed bands around 3500 cm^−1^ are attributed to the stretching vibration modes of the adsorbed water molecules and surface hydroxyl groups. After UV treatment, the intensity of these peaks decreases significantly that reveals the desorbing or dissociating of the hydroxyl groups from the device which in turn makes the surfaces of the device components less hydrophilic (Fig. 10). It is proposed that under UV irradiation, the adsorbed water molecules can be dissociated into the oxygen, hydrogen, and hydroxyl radicals that can passivate the dangling bonds in the perovskite crystals. As stated, in organic-inorganic halide perovskite, there are both negatively charged anionic and positively charged cationic defects, such as I^−^ and MA^+^ vacancies^15^. Dissociation of water molecules can provide the passivation of these defects by occupying vacancy sites to compensate the I^−^ and MA^+^ losses on the perovskite film surfaces, as explained in Fig. 9. The trapping of charge carriers at defect sites is detrimental for the device performance resulting in nonradiative recombination loss and deteriorates the carrier lifetime. Recently, different molecules have been employed as electron donors or acceptors to passivate the defects of perovskite crystals. The passivation molecules such as choline iodide and chloride^15^, pyridine, and thiophene^32^ interact with the charged defects and neutralize the related defect induced charge traps.

Further, light irradiation can cause the disappearance of surface hydroxyl groups that play a vital role during the photovoltaic process. The hydroxyl group can promote a nonradiative energy transfer from perovskite crystals to the O-H vibration states and impede the charge injection and transport^38,39^. Tan *et al*. also reported the desorption of OH species from the surface of TiO_2_ under the irradiation of UV light with different wavelength^40^. In fact, it causes the modification of TiO_2_/perovskite interface and subsequently charge transport which is confirmed by impedance spectroscopy.

## Conclusions

In summary, we reported a new facile, fast and low cost method for recovery of degraded perovskite devices based on UV-irradiation. In this method, not only the performance of the aged devices was fully recovered, but also the enhancement of the PCE was achieved. UV-treatment caused modifications in both bulk properties of the perovskite layer and interfaces. It was shown that UV-irradiation was highly desirable to electronically passivate the shallow and deep defects at grain boundaries and surfaces to boost the PCE, and to heal defects to prolong the durability of perovskite devices. As proposed, the defect passivation was occurred by dissociation of the adsorbed water molecules and hydroxyl groups under the UV-irradiation. Our findings provided an opportunity for defect passivation to prolong the durability and boost the PCE of PSCs.

## Experimental Section

### Materials and device fabrication

In order to fabricate perovskite solar cell, fluorine doped tin oxide (FTO) coated glass substrates are etched and cleaned with deionized water, acetone, and isopropanol, respectively. After drying of substrates at 100 °C for 30 min, they aree coated with a compact layer of TiO_2_ by spin coating a mild acidic solution of tetraisopropyl orthotitanate in ethanol followed by annealing at 500 °C for 30 min. The compact layer is treated by TiCl_4_ solution and annealed at 500 °C for 30 min. Then, the mesoporous TiO_2_ layer is deposited by spin coating of TiO_2_ paste at 5000 RPM for 30 s, dried at 70 °C for 30 min, and annealed at 500 °C, and finally is treated by TiCl_4_. Perovskite layer is deposited via two-step process from PbI_2_ precursor solution (1 M in anhydrous DMF) and CH_3_NH_3_I solution (7 mg/ml in anhydrous IPA). P3HT is used as hole transporting layer (HTL) that is spin coated from 10 mg/ml solution of P3HT in chlorobenzene. Finally, a 100 nm-thick Au layer is deposited on the top of the P3HT film by thermal evaporator (Nanostructure Coating Co. Iran) in vacuum condition (~10^−5^ torr), yielding an active area of 0.11 cm^2^.

### Characterization

The morphology and structure of the films are characterized using Phenom scanning electron microscopy (SEM) and FESEM (TESCAN). Besides, Veeco atomic force microscopy (CP-Research, USA) is used to take perovskite layer surface topography images. The absorption and transmission spectra of the device layers are recorded by an Avantes UV-Visible spectrophotometer (AvaSpec 2048, Netherland). In order to investigate the crystalline nature of perovskite films, X-ray diffraction (XRD) spectra are obtained on a Philips diffractometer (model: X’Pert MPD) equipped with a proportional Xe filled detector, Cu tube (*λ* = 1.54056 Å). Current-voltage (I-V) characteristics of the fabricated devices are measured by an Ivium stat potentiostat (XRE model, Netherland) under a calibrated AM 1.5 solar simulator at 100 mW/cm^2^ light intensity (Sharif Solar 10–2, Iran). ^1^H nuclear magnetic resonant (NMR) spectroscopy is performed using a BRUKER 500 MHz spectrometer. FTIR spectra of precursors and perovskite films are acquired using Perkin-Elmer spectrometer (Frontier). IVIUM stat potentiostate/galvanostate (XRE model, Netherland) is used to record impedance spectra of the devices in the frequency range of 1 to 100 KHz under light and bias V = 0 V. To measure capacitance-voltage characteristics, a DC voltage is applied under dark condition and the AC perturbation is 10 mV at 10 kHz.

## Supplementary information

High Power UV-Light Irradiation as a New Method for Defect Passivation in Degraded Perovskite Solar Cells to Recover and Enhance the Performance
