# Supplementary material for: High Power UV-Light Irradiation as a New Method for Defect Passivation in Degraded Perovskite Solar Cells to Recover and Enhance the Performance
Source: Sci Rep. 2019 Jul 1;9:9448. doi: 10.1038/s41598-019-45756-1 (PMC6603019; doi:10.1038/s41598-019-45756-1)
Supplement: Supplementary file 1 — High Power UV-Light Irradiation as a New Method for Defect Passivation in Degraded Perovskite Solar Cells to Recover and Enhance the Performance [file 41598_2019_45756_MOESM1_ESM.docx]

Supporting Information

High Power UV-Light Irradiation as a New Method for Defect Passivation in Degraded Perovskite Solar Cells to Recover and Enhance the Performance

*Farzaneh Arabpour Roghabadi ^1^, Nasibeh Mansour Rezaei Fumani^2^, Maryam Alidaei^3^, Vahid Ahmadi^1*^, Seyed Mojtaba Sadrameli^2*^*

E-mail: [v_ahmadi@modares.ac.ir](mailto:v_ahmadi@modares.ac.ir), [sadrameli@modares.ac.ir](mailto:sadrameli@modares.ac.ir)

Keywords: Perovskite solar cell, recovery, UV-light irradiation, degraded, defect passivation

**UV-treatment method**

To treat the PSK devices, the device is illuminated by UV-light source with a power of 400 and 1000W in a stainless steel box. The illumination time is changed between 30s to 10 min. Samples are exposed to UV light from the Au contact side. Device to lamp distance is maintained at 20 cm.

**Figure S 1 Hysteresis behavior of the perovskite device before and after UV-treatment. The device is stored in ambient condition for 35 days.**

Table S 1 Normal device performance before and after UV-treatment with 400 and 1000 W UV-Light.

| **Device** | **V­_OC_**  **(V)** | **J_SC_ (mA/cm^2^)** | **FF**  **(%)** | **PCE**  **(%)** |
| --- | --- | --- | --- | --- |
| **UV-400W** | | | | |
| **Fresh device** | 0.85 | 19.5 | 60.1 | 10 |
| **Aged device** | 0.83 | 13.1 | 58 | 6.3 |
| **Treated device** | 0.88 | 20.1 | 62.5 | 11 |
| **UV-1000W** | | | | |
| **Fresh device** | 0.86 | 19.7 | 59.8 | 10.13 |
| **Aged device** | 0.86 | 12.14 | 56.9 | 5.9 |
| **Treated device** | 0.9 | 22 | 61.5 | 12.1 |

Table S 2 Performance of HTM free devices before and after UV-treatment.

| **Device** | **V­_OC_**  **(V)** | **J_SC_ (mA/cm^2^)** | **FF**  **(%)** | **PCE**  **(%)** |
| --- | --- | --- | --- | --- |
| **Fresh device** | 0.75 | 5.5 | 50 | 2.0625 |
| **Aged device** | 0.73 | 4 | 49 | 1.4 |
| **Treated device** | 0.79 | 7 | 58 | 3.2074 |


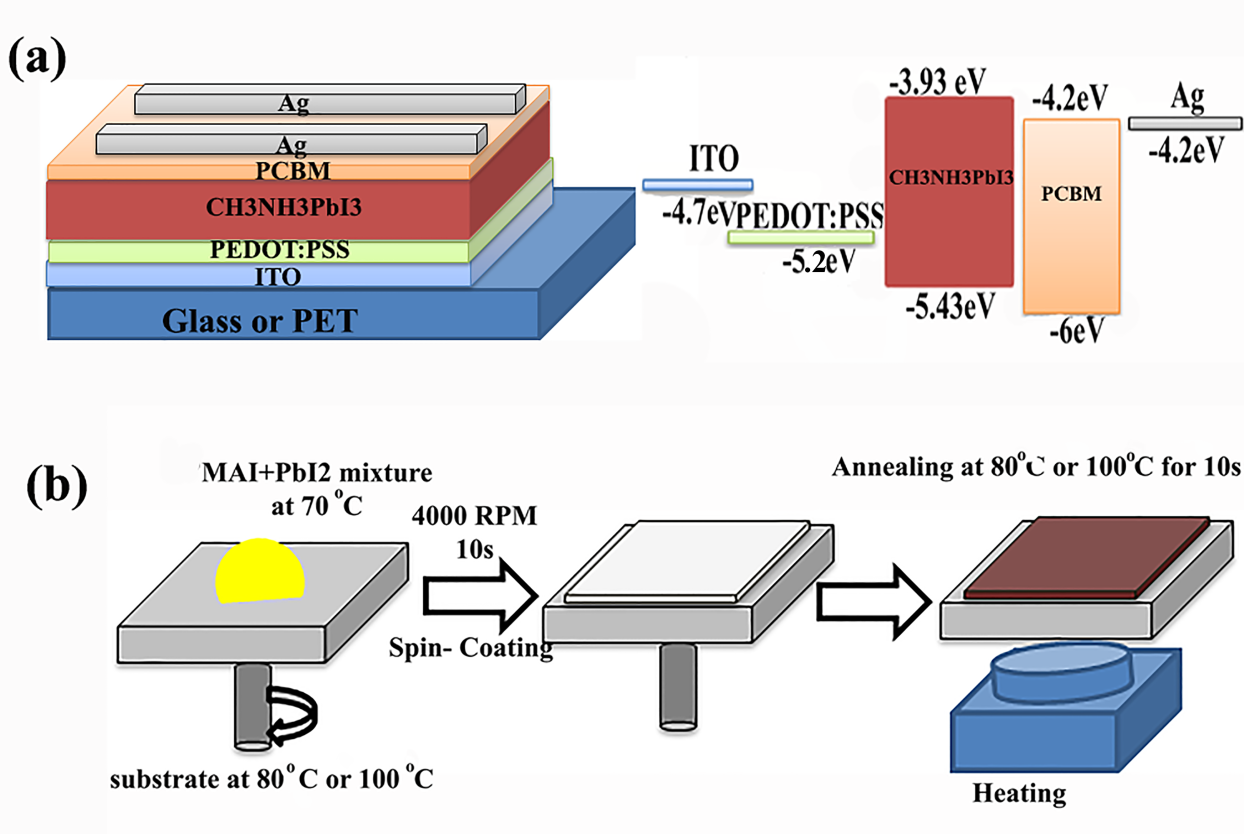


Figure S 2 Schematic structure of the normal perovskite solar cells and the energy diagram of the inverted device components.

**Figure S 3** NMR spectrum of P3HT layer after UV-treatment for 10 min.

Table S 3 Performance of inverted devices before and after UV-treatment.

| **Device** | **V­_OC_**  **(V)** | **J_SC_ (mA/cm^2^)** | **FF**  **(%)** | **PCE**  **(%)** |
| --- | --- | --- | --- | --- |
| **Fresh device** | 0.93 | 18.2 | 59 | 10 |
| **Aged device** | 0.85 | 16.8 | 56 | 8 |
| **Treated device** | 0.94 | 18.9 | 61.5 | 11 |

Table S 4 Performance of fresh normal devices before and after UV-treatment.

| **Device** | **V­_OC_**  **(V)** | **J_SC_ (mA/cm^2^)** | **FF**  **(%)** | **PCE**  **(%)** |
| --- | --- | --- | --- | --- |
| **Fresh device** | 0.9 | 19.8 | 62 | 11 |
| **Treated device** | 0.91 | 20 | 63 | 11.5 |


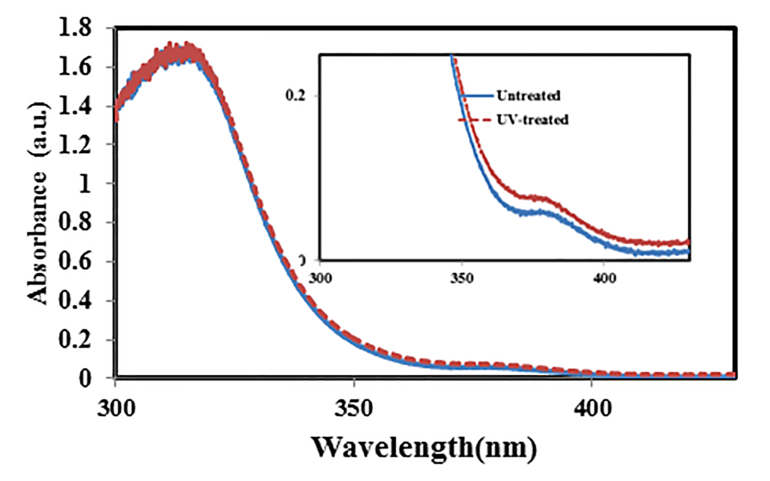


**Figure S 4 Absorbance of TiO_2_ layer deposited on FTO substrate before and after UV-treatment.**


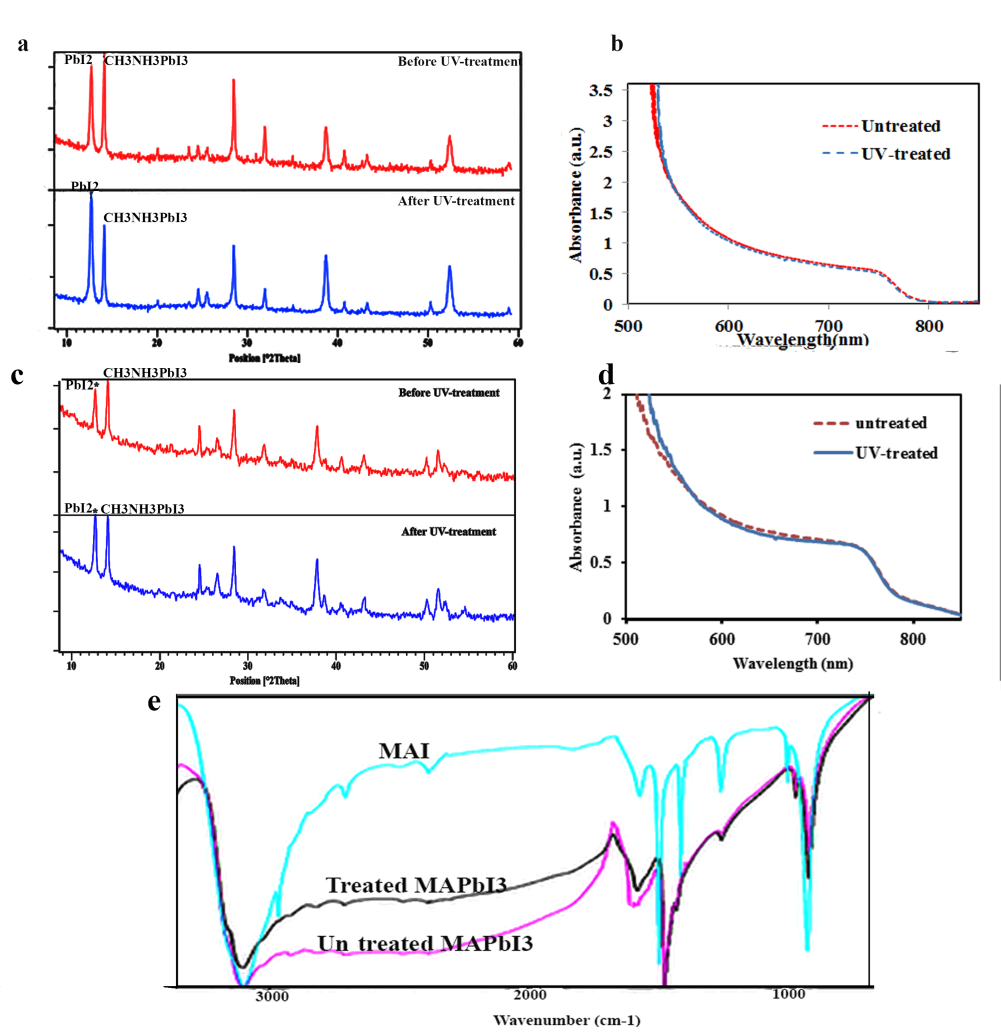


**Figure S 5 (a) XRD spectra of CH_3_NH_3_PbI_3_ layer deposited on FTO substrate, (b) absorbance spectra of CH_3_NH_3_PbI_3_ layer deposited on FTO substrate, (c) XRD spectra of CH_3_NH_3_PbI_3_ layer deposited on FTO/c-TiO_2_/m-TiO_2,_ (d) absorbance spectra of CH_3_NH_3_PbI_3_ layer deposited on FTO/c-TiO_2_/m-TiO_2_, (e) FTIR spectra of CH_3_NH_3_PbI_3_ layer before and after UV-treatment.**

Figure S 6 PL spectra of perovskite layer deposited on glass before and after UV-treatment, λ_ex_=600 nm.


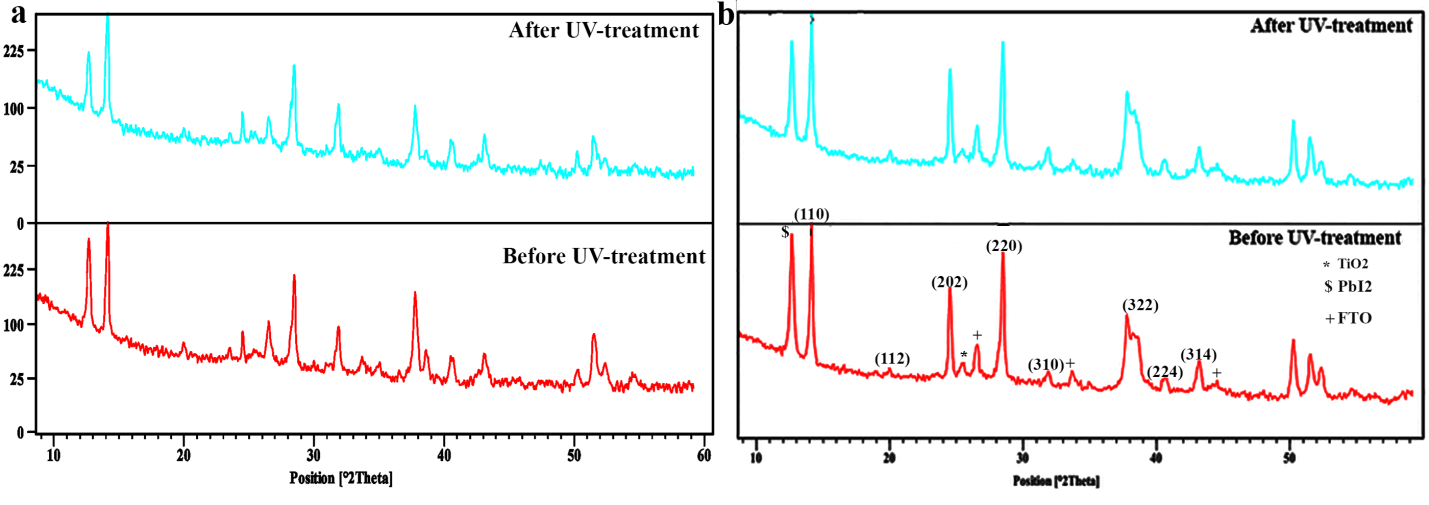


Figure S 7 (a) XRD spectra of FTO/c-TiO_2_/m-TiO_2_/CH_3_NH_3_PbI_3_/P3HT multilayer, (b) XRD spectra of complete device with FTO/c-TiO_2_/m-TiO_2_/CH_3_NH_3_PbI_3_/P3HT/Au structure before and after UV-treatment.

Figure S 8 UV-transmittance of a 100 nm-Au thin film deposited on glass.

Figure S 9 UV-reflectance of a 100 nm-Au thin film deposited on glass (red), a 100nm-Au thin film deposited on Glass/FTO/c-TiO_2_/m-TiO_2_/Perovskite/P3HT multilayer (blue).

Figure S 10 Capacitance spectra of the device as a function of applied bias.
